# Eclampsia and Postpartum Depression in the Setting of Recurrent Prenatal COVID-19

**DOI:** 10.7759/cureus.29654

**Published:** 2022-09-27

**Authors:** Olivia M Cook, Sahar Zargar, Wanda Torres

**Affiliations:** 1 Medicine, Nova Southeastern University Dr. Kiran C. Patel College of Osteopathic Medicine, Clearwater, USA; 2 Obstetrics and Gynecology, Nova Southeastern University Dr. Kiran C. Patel College of Osteopathic Medicine, Clearwater, USA

**Keywords:** pregnancy, postpartum depression, covid-19, gestational hypertension, pre-eclampsia, eclampsia

## Abstract

Current research suggests COVID-19 in pregnancy is associated with poor maternal and fetal outcomes, although the exact mechanisms remain unclear, and the approach to the management of affected patients presents a distinct challenge to clinicians. We present a case of gestational hypertension, eclampsia, and postpartum depression in a 39-year-old gravida 4, para 0030 (G4P0) pregnant patient following multiple prenatal severe acute respiratory syndrome-related coronavirus 2 (SARS-CoV-2) infections. After a case of coronavirus disease-19 (COVID-19) during her first trimester, the patient received a two-dose mRNA vaccine against SARS-CoV-2. Despite vaccination, she again contracted COVID-19 during her third trimester of pregnancy. She subsequently developed gestational hypertension at 38 weeks necessitating a cesarean section at 38+4 weeks. The patient delivered a healthy neonate, however, her postpartum course was complicated by eclampsia and postpartum depression. This case bolsters current literature and emphasizes the necessity of continued research into the effects of COVID-19 in pregnant and postpartum women.

## Introduction

According to the World Health Organization (WHO), the coronavirus disease-19 (COVID-19) global pandemic has led to > 500 million confirmed infections and > 6 million deaths worldwide [1]. Pregnant women comprise a unique population of individuals vulnerable to severe acute respiratory syndrome-related coronavirus 2 (SARS-CoV-2) infection due to the complex physiologic and immunologic changes that occur during pregnancy [2,3]. Infection in pregnant women is associated with an increased risk of prenatal complications and maternal morbidity and mortality [4]. The recent INTERCOVID Multinational Cohort Study demonstrated increased rates of preeclampsia, eclampsia, and hemolysis, elevated liver enzymes, and low platelet count (HELLP) syndrome in women with COVID-19 in pregnancy. Women with preexisting obesity, diabetes, hypertension, and chronic cardiac and respiratory diseases with concurrent SARS-CoV-2 infection were found to have a four times greater risk of developing preeclampsia and eclampsia [5]. However, there remains a limited number of case studies communicating the adverse effects of COVID-19 in pregnancy, and there are even fewer reported cases that explore the sequelae of SARS-CoV-2 infection in the peripartum period.

The emergence of the COVID-19 pandemic has amplified mental disorders globally, with an estimated 27.6% and 25.6% increase in major depressive disorder and anxiety disorder, respectively [6]. Pregnancy is associated with an increased risk of depression and anxiety [7]. An estimated 12% of otherwise healthy women experience postpartum depression, however recent studies have suggested a 22% prevalence of postpartum depression in connection to the COVID-19 pandemic [8,9]. Moreover, prenatal and postnatal mental disorders are associated with physical diseases, including gestational hypertension and preeclampsia [10].

Understanding the relationship between COVID-19 and maternal morbidity and mortality has presented a challenge to researchers and clinicians, emphasizing the importance of presenting complex COVID-19 cases associated with adverse outcomes of pregnancy. We report a case of a vaccinated pregnant woman with multiple episodes of COVID-19 followed by gestational hypertension. After delivering a healthy neonate via cesarean section at 38+4 weeks, her pregnancy course was further complicated by postpartum eclampsia and postpartum depression.

## Case presentation

A 39-year-old gravida 4, para 0030 (G4P0) woman at 7 weeks gestation developed fever and myalgia. She had no other symptoms and denied SARS-CoV-2 exposure. A nucleic acid amplification test (NAAT) resulted positive for SARS-CoV-2, but the patient described an overall mild infection and recovered in self-isolation. Prior to the infection, her prenatal risk factors included advanced maternal age (AMA) and class 1 obesity (BMI 32.6 kg/m2), and her only medications were prenatal vitamins with iron and low-dose aspirin for preeclampsia prevention. She was scheduled for a cesarean section under general anesthesia at 39 weeks gestation due to a history of lumbar spinal fusion and stabilization with orthopedic hardware. The patient had an extensive history of back surgeries secondary to military injuries incurred, thus the patient’s orthopedic surgeon advised cesarean delivery under general anesthesia to prevent potential compromise of her hardware.

She was later vaccinated for SARS-CoV-2 with the Moderna two-dose vaccine at 15+1 weeks and 19+1 weeks. Despite vaccination and self-reported mask-wearing and social distancing, she developed a productive cough at 29+5 weeks on July 27, 2021 after a confirmed SARS-CoV-2 workplace exposure. She reported working as a gym manager. The patient presented to urgent care at the onset of symptoms. Her vitals were stable with a temperature of 36.7 °C, heart rate of 91 beats per minute, blood pressure 120/79 mm Hg, respiratory rate of 16 breaths per minute, and oxygen saturation of 97% on room air. SARS-CoV-2 NAAT resulted positive. Her spouse developed similar symptoms and also tested positive for SARS-CoV-2. The patient was prescribed three days of oral guaifenesin 400 mg/codeine 40 mg/day as needed for severe cough, however, she declined treatment with dexamethasone. Additionally, she began taking vitamin C 1000 mg/day, vitamin D3 4000 IU/day, zinc 30 mg/day, and lavender oil. Her symptoms improved initially, but one week later the cough worsened, and she developed fatigue. She denied dyspnea, fever, headache, and chills. She was prescribed another three-day course of guaifenesin/codeine, and she continued taking vitamin C, vitamin D, and zinc until her symptoms completely resolved. An anatomy ultrasound demonstrated normal development and appropriate growth without evidence of intrauterine growth restriction (IUGR).

At 34 weeks gestation, the patient reported two more workplace exposures to SARS-CoV-2, less than one month after her prior infection. She developed cough and wheezing, however, she was not re-tested for SARS-CoV-2 and did not require treatment.

At 38+4 weeks gestation, the patient presented to the clinic with a headache unrelieved by acetaminophen and serial blood pressure readings of 142/96 mm Hg, 146/102 mm Hg, and 156/104 mm Hg over four hours. Urine protein was not obtained, however, the patient’s hypertension and neurologic symptoms established a diagnosis of preeclampsia. Despite symptoms, fetal heart rate tracing displayed a category 1 pattern. The patient was sent to labor and delivery and underwent a cesarean delivery under general anesthesia, per the advice of her orthopedic surgeon, for the management of preeclampsia. She gave birth to a healthy 6 lb 5 oz female neonate with Apgar scores of 8 and 9 at 1 and 5 minutes, respectively. Elevated blood pressure and associated symptoms improved with delivery. The mother and infant were discharged on postoperative day 3 with a blood pressure of 130/76 mm Hg. Several days following discharge, she began noticing progressive lower extremity edema and headaches. One week postpartum, her blood pressure increased to 150/90 mm Hg, and she experienced a severe headache with visual disturbances followed by an eclamptic seizure of unknown duration. She was admitted to the hospital, and intravenous (IV) magnesium sulfate was initiated and maintained for 24 hours, but she did not require any anti-hypertensive medications. A head computed tomography (CT) was unremarkable. She was discharged after 2 days with blood pressures between 120-130/70-80mm Hg. At two weeks postpartum, the patient reported complete resolution of symptoms and blood pressure was 120/80 mm Hg.

**Figure 1:**
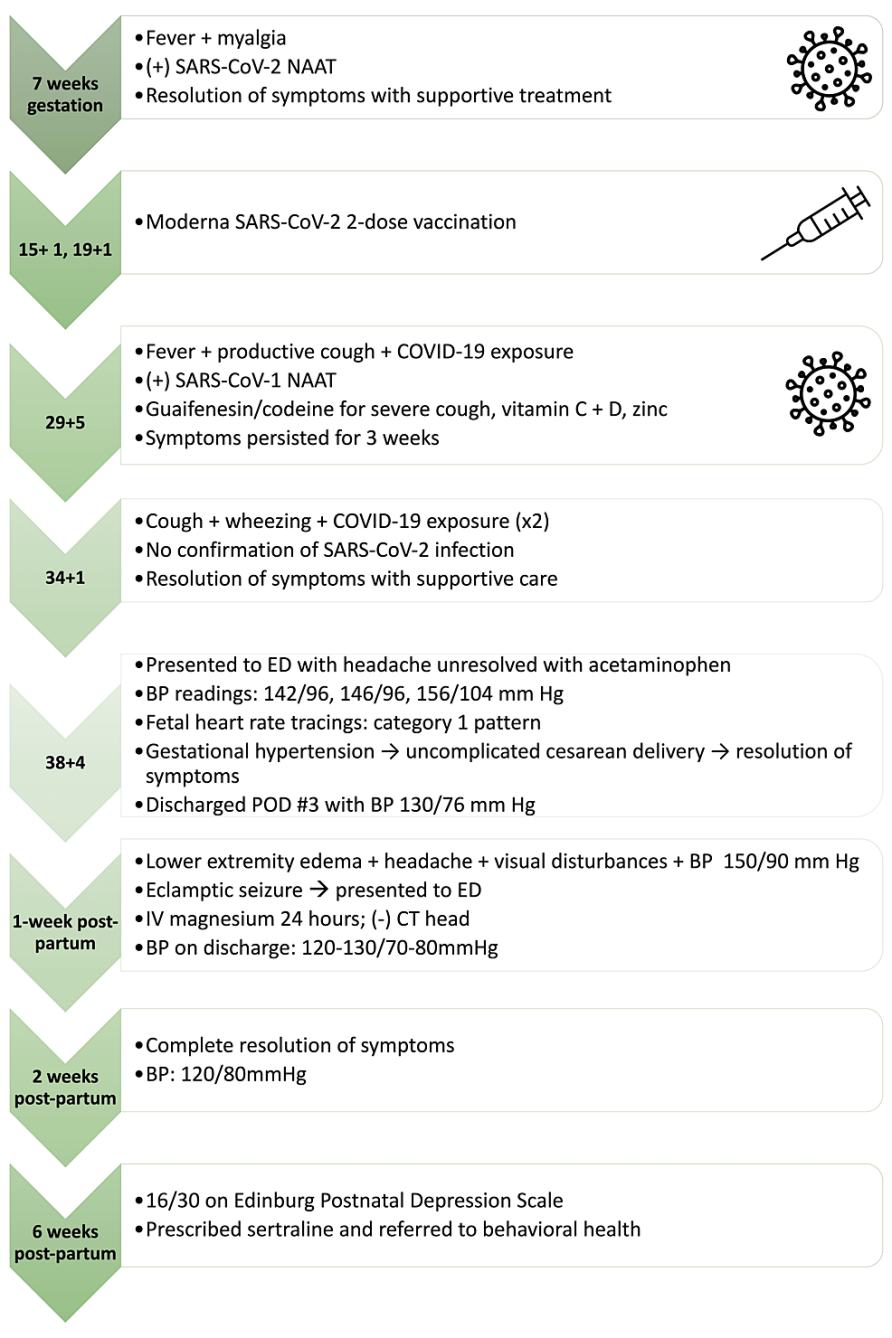
Timeline of Clinical Course SARS-CoV-2: Severe acute respiratory syndrome coronavirus-2; NAAT: Nucleic acid amplification test; COVID-19: Coronavirus disease 2019; ED: Emergency department; BP: Blood pressure; mm Hg: Millimeters of mercury; POD: Post-operative day; IV: Intravenous; CT: Computed tomography scan

At six weeks postpartum, the patient scored a 16 on the Edinburgh Postnatal Depression Scale. There were no indications of homicidal or suicidal behavior, but she displayed a tearful affect and significant mental distress. She reported difficulty sleeping and little support at home due to her spouse's full-time job and her friends not being vaccinated against SARS-CoV-2. The patient was diagnosed with major depressive disorder with postpartum onset and started on sertraline 25 mg/day and referred to behavioral health. At the two-month follow-up, the patient reported improved mood and sleep and decreased depression and anhedonia (Figure 1).

## Discussion

We describe a vaccinated pregnant patient with multiple SARS-CoV-2 infections and subsequent peripartum physical and mental sequelae. The case supports existing research proposing an association between COVID-19 and adverse outcomes of pregnancy and endorses continued research into the effects and management of COVID-19 in pregnancy and postpartum. Given that COVID-19 vaccination in pregnant women has been shown to elicit a comparable immune response to that of non-pregnant controls with antibody production as early as five days after the first dose, this patient’s reinfections highlight the necessity of close monitoring regardless of vaccination status [11,12]. Underlying medical conditions such as obesity and AMA, as seen in this patient, increase the risk for SARS-CoV-2 infections, sequelae, and other complications in pregnancy such as hypertensive disorders [13]. Furthermore, the patient’s elevated occupational exposure to COVID-19 may have contributed to her recurrent SARS-CoV-2 infections.

While this patient’s development of preeclampsia and eclampsia may be a result of her comorbidities, the overlapping dysregulatory mechanisms of SARS-CoV-2 and hypertensive disorders of pregnancy on the renin-angiotensin system (RAS) suggest a plausible pathway by which SARS-CoV-2 infection could elicit or contribute to the development of eclampsia [14,15]. RAS plays an important role in maintaining blood pressure, fluid balance, and placental function during pregnancy [14]. Angiotensin-converting enzyme 2 (ACE2,) a key regulator of RAS, converts angiotensin (Ang) II to Ang-(1-7,) thereby protecting against the vasoconstrictive and proinflammatory effects of Ang II [16]. In an uncomplicated pregnancy, both ACE2 and Ang-(1-7) have been shown to be upregulated in the placental syncytiotrophoblast, cytotrophoblast, and villous stroma [16,17]. Conversely, an elevated Ang II to Ang-(1,7) ratio is associated with preeclampsia and other adverse outcomes of pregnancy [15]. 

The ACE2 receptor also serves as the target receptor for SARS-CoV-2; the binding and entry of the virus not only leads to infection but also leads to dysregulation of RAS. Specifically, it causes downregulation of Ang-(1-7,) resulting in vasoconstriction, inflammation, end-organ damage, and increased coagulation, effects that similarly occur in preeclampsia and eclampsia [13-15]. Studies show that placentas infected with SARS-CoV-2 result in alteration of placental RAS and increased levels of soluble fms-like tyrosine kinase-1 (sFlt1), a signatory marker of preeclampsia [16]. Although the etiological association between SARS-CoV-2 and hypertensive disorders of pregnancy remains unclear, there is significant overlap between their dysregulatory effects on RAS which necessitates additional research.

The postpartum period poses numerous challenges, including hormonal changes, sleepless nights, and the new responsibility of caring for a newborn [7]. Risk factors for postpartum depression include depression or anxiety during pregnancy, recent stressful life events, poor social support, and a history of depression [18]. Unsurprisingly, social relationships have been shown to improve psychological well-being and provide access to resources during stressful periods and transitions in life, such as pregnancy and child-rearing [19].

Preliminary studies and surveys suggest that rates of anxiety, depression, post-traumatic stress disorder (PTSD), and loneliness in perinatal women have increased with the emergence of COVID-19 [20,21]. Concerns of viral transmission to the mother or newborn can result in self-isolation, preventing social support that is protective against the development of postpartum depression [7,20]. Unsurprisingly, our patient developed postpartum depression following a pregnancy complicated by two confirmed episodes of COVID-19, eclampsia, and isolation from family and friends.

Despite the importance of this case, several limitations were encountered. For example, it’s unclear if the patient’s third episode of respiratory illness was caused by another COVID-19 infection. Furthermore, at the time she developed hypertension, a urine protein was not obtained, a test that would have better established a diagnosis of preeclampsia. Additionally, a pathologic analysis of the patient’s placenta would have allowed examination of the potential association between COVID-19 and hypertensive disorders of pregnancy.

## Conclusions

This case emphasizes the importance of perinatal care and the evaluation of both physical and mental health in obstetric patients, especially during pandemics and other societal crises. There is a potential interplay between COVID-19 and hypertensive disorders of pregnancy that must be further researched. The negative impact of pandemics on the mental health of society has been well established, however, improving access and approach to care for vulnerable populations, such as pregnant or postpartum women, is needed.
